# Bob-1 is expressed in classic Hodgkin lymphoma

**DOI:** 10.1186/1746-1596-2-10

**Published:** 2007-03-08

**Authors:** Howayda S Abd El All

**Affiliations:** 1 Department of Pathology, Faculty of Medicine, Suez Canal University, Ismailia, Egypt

## Abstract

**Background:**

Almost all researchers agree on the lack of Bob-1 expression in Hodgkin/Reed-Sternberg (H/RS) cells in classic Hodgkin lymphoma (CHL), and utilize this marker as a diagnostic tool in conjunction with other markers to differentiate between lymphocyte predominance Hodgkin lymphoma (LPHL) and CHL.

**Aim:**

To study the immunohistochemical (IHC) expression of Bob-1 in Egyptian CHL and to correlate this expression with Epstein-Barr virus (EBV) viral load.

**Materials and methods:**

Paraffin sections of randomly selected 18 CHL cases were included: 2 lymphocyte rich (LR), 4 mixed cellularity (MC), 10 nodular sclerosis (NS) and 2 lymphocyte depletion (LD). All cases were immunostained for Bob-1. EBV was evaluated by EBV early RNA transcripts in situ hybridization (EBER ISH) and immunostaining for EBV latent membrane protein-1 (LMP-1).

**Results:**

Sixty seven percent of cases (12/18) were positive for EBV by ISH and/or immunostaining for LMP-1. Moderate to strong nuclear Bob-1 was observed in 94% of cases. The positivity ranged between 25–100%. Bob-1 immunoreactivity was strongly associated with EBV positivity (p < 0.001).

**Conclusion:**

This study proves nuclear IHC expression of Bob-1 on H/RS in CHL implying the difficulties in applying this marker to differentiate between LPHL and CHL. Does this difference between Western and Egyptian CHL reflect genetic and/or environmental factors, or simply no difference exists as most researchers are concentrated on the Western population and no comparative studies have been done. Studies from other countries might answer this question.

## Background

According to the World Health Organization (WHO) classification of haematological malignancies [1], the B-cell specific transcriptional co-activator or Bob-1/OCA-B is not expressed on H/RS cells, a point used to differentiate between CHL and LPHL, the latter being Bob-1 positive. Bob-1/OCA-B located on chromosome 11q23.1 [2], is involved in the transcription of immunoglobulin (Ig) genes through recruitment of the highly conserved octamer site of Ig promoters, mediated by either Oct-1 or Oct-2 transcription factor [3]. Bob-1 is essential for the response of B cells to antigens, and is required for the formation of the germinal centre (GC) [4].

EBV, the main etiologic agent for HL [5], has been reported in 30–50% of cases in developed countries and in up to 95% of cases in developing ones [6]. The detection of EBV in H/RS cells is mainly based on the detection of the latently expressed gene LMP-1 or on the detection of EBV early RNA transcripts (EBER) [7].

As H/RS cells originate from the GC [8-10], they are expected to express B cell markers such as Bob-1. Therefore this work was undertaken to study the expression of Bob-1 in Egyptian CHL and to correlate this expression with EBV viral load in an attempt to find out if differences do exist between Western and Egyptian CHL, for optimal assessment of the treatment regime.

## Materials and methods

### Classic Hodgkin lymphoma cases

Paraffin sections of randomly selected CHL were classified according to the WHO [1]. A total of 18 CHL cases were studied. These included 2 cases of LRHL, 4 MCHL, 10 NSHL, 2 LDHL. The positive controls consisted of 2 reactive nodes selected with follicular hyperplasia and 2 cases of LPHL. The initial diagnostic panel included CD30, CD15, CD20 and CD3. EMA and ALK-1 were performed whenever indicated to rule out LPHL and anaplastic large cell lymphoma.

### Immunohistochemistry

The expression of Bob-1 and EBV LMP-1 was evaluated by immunostaining (table 1). Following deparaffinization, endogenous peroxidase was inhibited by tissue sections incubation for 10 minutes at room temperature in 0.3% H2O2. Following antigen retrieval, slides were rinsed in distilled water and finally phosphate buffered saline (PBS). All incubations were performed at room temperature. After incubation with the primary antibody, sections were rinsed in PBS and incubated with the LSAB-2 detection kit and the steps were followed according to the manufacturer instructions (Dakocytomation). Diaminobenzedine tetrachloride (DAB) was applied for 10 minutes and lastly, sections were counterstained with Harris haematoxylin (Hx).

**Table 1:** Reagents used for IHC in the study

	Bob-1	LMP-1
Source	Santa Cruz	Dakocytomation
Reference	Sc 955 rabbit polyclonal	M0897, monoclonal
Heat induced epitope retrieval	Citrate pH 6.0, 750 w × 5 m × 3 times	
Dilution	1:1500	1:50
Incubation	60 m	30 m
Interpretation	Nuclear	Membranous, cytoplasmic and/or paranuclear dot

### Evaluation of immunohistochemical staining

The percentage of positively stained H/RS cells were semi-quantitatively determined as follows: 0- absence or staining of less than 5% of H/RS cells; +) 5–25%; ++) > 25–50%; +++) > 50–75%; ++++) > 75% of H/RS cells showed a positive staining. In all cases, small reactive lymphocytes served as positive internal control for Bob-1, in addition to the positivity of the reactive nodes and lymphocytic/histiocytic cells of LPHL.

### In-situ hybridization

The ISH steps were performed according to the manufacturer's instructions (Novocastra, NCL-EBV-K). The slides were first dewaxed in xylene, hydrated in descending grades of alcohol and lastly immersed in water. One hundred μl of proteinase K in 0.05 mM Tris/HCL buffer pH 7.6 were applied for 10 minutes at 37°C. This step was followed by slides immersion in water, dehydration and air drying. Depending on the tissue section, 20 to 50 μl of the probe hybridization solution were applied. Sections were coverslipped and incubated for 2 hours at 37°C. The covers were allowed to drain off into a beaker; they were then washed in Tris containing 0.1% triton X-100. For the detection, 100 μl of the blocking solution was applied for 10 minutes followed by rabbit F (ab') anti-FTTC conjugated to alkaline phosphatase (AP) diluted 1:100 for 30 minutes. Slides were subsequently washed in TBS followed by the AP substrate buffer. The AP activity was demonstrated by incubation in dark overnight with a mixture solution of 5 bromo-4 choro indolyl phosphate, nitro-blue tretrazolium (BCNT). Finally, the slides were washed and counterstained with Mayer's haematoxylin. The control of the procedure included positive control sections and negative control probe supplied with the ISH kit.

### Evaluation of in-situ hybridisation

The staining was considered positive when dark blue to black dots were encountered in the nuclei of the H/RS cells together with positivity of the positive control tissue and negativity of the duplicate sections hybridized with the negative probe.

### Statistical analysis

The association between Bob-1 positive cells and EBV viral load was evaluated using the Chi square test.

## Results

In reactive nodes, residual follicles and inflammatory milieu of HL, strong nuclear Bob-1 was expressed in GC B cells while moderate staining was seen in scattered mantle zone B cells and interfollicular T cells.

**Figure 1:**
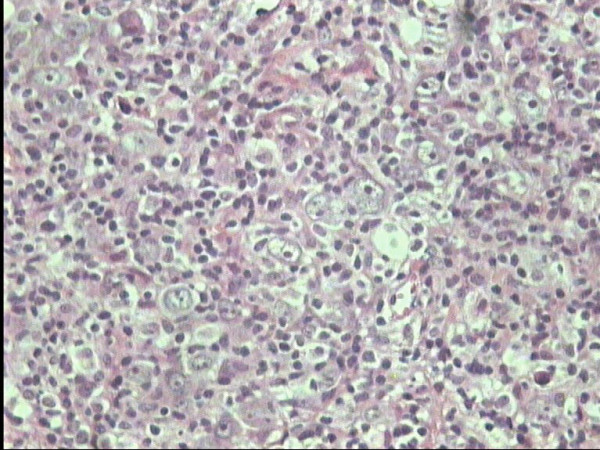
MCHL H/RS cells in an inflammatory background, H&E × 40.

**Figure 2:**
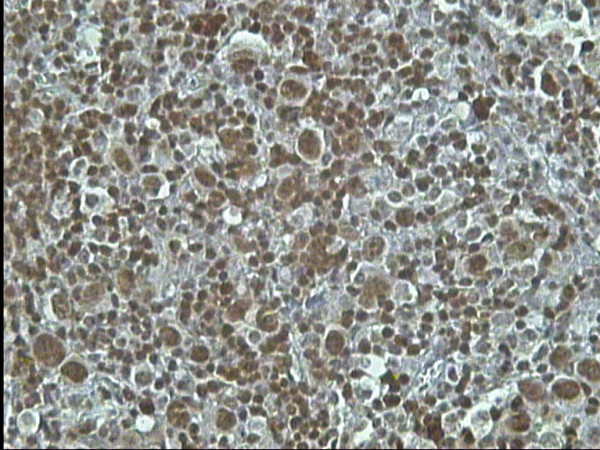
MCHL: strong nuclear Bob-1 staining in all H/RS cells, Bob-1 immunostaining, DAB, Hx, × 40.

**Figure 3:**
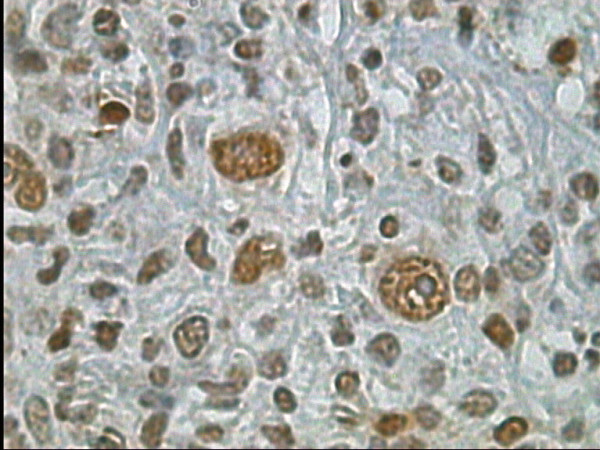
MCHL: higher power magnification of the previous figure, Bob-1 immunostaining, DAB, Hx, ×100.

**Figure 4:**
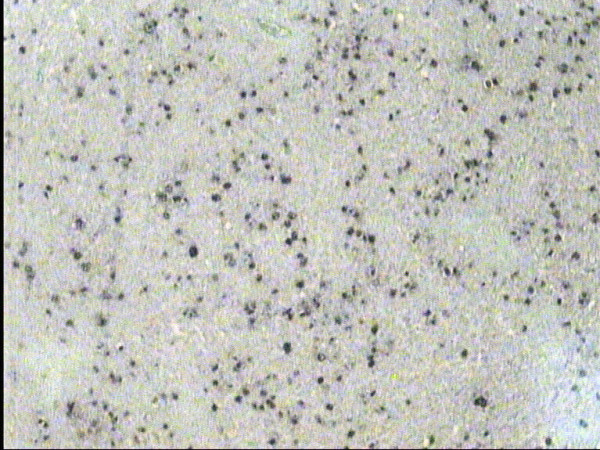
MCHL: strong nuclear EBV staining, EBER-ISH, BNCT, Hx, × 20.

**Figure 5:**
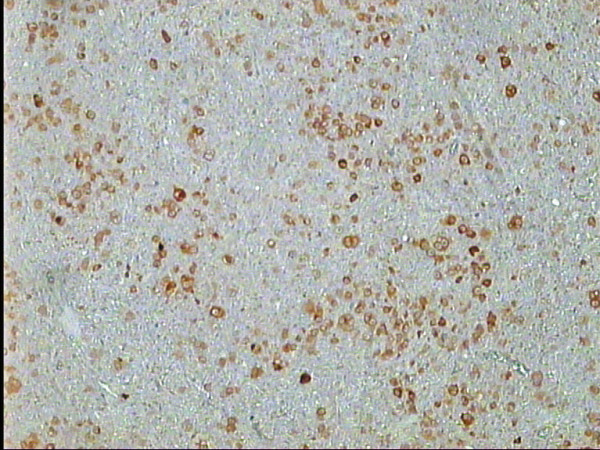
MCHL: LMP-1 immunostaining of the same case, DAB, Hx, × 20.

In CHL, moderate to strong nuclear IHC Bob-1+ H/RS cells were encountered in 94% of cases irrespective of the subtype. The percentage of positive cells ranged from 25–100% (table 2, figures 1, 2, 3). Membranous, cytoplasmic and/or paranuclear dot staining for LMP-1 (figure 4) was identified in 61%, while nuclear staining for EBER-ISH (figure 5) was encountered in 67% of cases. A close association was found between Bob-1 immunoreactivity and EBV viral load (p < 0.001).

**Table 2:** Bob-1 and EBV expressions in CHL

CHL Subtype	Bob-1	EBV viral load	
		
		EBER	LMP-1
LRHL	2/2	2/2	1/2
MC	4/4	3/4	3/4
NS	9/10	6/10	6/10
LD	2/2	1/2	1/2
Total	17/18	12/18	11/18
%	94%	67%	61%

## Discussion

CHD is a B-cell neoplasm in nearly all instances derived from the GC B cells harbouring somatically mutated IgV region genes. However, these cells have consistently lost their Ig gene transcription ability, due to functional defects in the Ig gene regulatory elements [10-12]. The defect has been attributed to crippling mutations [11,12], a defect in the transcription machinery due to lack of expression of the octamer transcription factor Oct2 and/or its coactivator Bob-1 [12,14], or epigenetic silencing in the inhibition of IgH transcription [15].

The expression of Bob-1 in H/RS cells was a surprising finding. To our knowledge, this is the first study reporting strong nuclear Bob-1 in almost all H/RS cells. In reactive nodes and non neoplastic cells in HL, Bob-1 IHC expression is concordant with the literature [16,17]. However, in neoplastic conditions, Bob-1 positivity is restricted to LPHL and has been a useful tool in differentiating it from CHL [13,18-20]. Only one previous study using tissue microarray, reported Bob-1+ H/RS cells in 17% of cases with strong Bob-1 positivity in only 2% of cases [21].

Should the strong IHC expression of Bob-1 in H/RS cells is really to be an unexpected finding? First, in HL cell lines, Oct-2 has been reported in one study to be constantly expressed on H/RS cells [22]. Second, a close resemblance has been found between primary mediastinal B-cell lymphoma (PMBL) and CHL [23-25]. PMBL signature genes revealed an extraordinarily robust gene expression relationship between PMBL and HL, strongly supporting a pathogenetic relationship between these two lymphoma types [24,25]. On IHC basis, PMBL are PAX5/BSAP+, Bob-1+, Oct-2+, PU.1+, Bcl-2+, CD30+, HLA-DR+, Bcl-6+/-, Mum-1+/- [26], markers already identified on CHL with the exception of PU.1, Bob-1 and Oct-2 [13,19,28,27]. In addition, the MAL protein initially a PMBL marker [29], has been identified on H/RS cells from a case of NSHL in the study of Rosenwald et al [24]. Furthermore, PMBL and HL have rearranged Ig genes but lack surface Ig [10,11,24]. Considering these previous findings together with the results of our research, one can conclude that the expression of Bob-1 in the Egyptian population is not an uncommon finding.

What could be the other explanations of Bob-1+H/RS cells in the present study? It has been speculated that EBV contributes to the transformation and maintenance of H/RS cells, by rescuing them from apoptosis. This has been attributed to the oncogenic potential of LMP-1 on B cells through upregulation of anti-apoptosis genes including bcl-2 [30], downregulation of p16INK4a [31] and activation of NFκB [32]. What is the relation between EBV and Bob-1? NFκB and Bob-1 are transcription factors required for mouse B cell differentiation, serum IgM production, late B cell maturation and function [33]. It seems that the activation of NFκB in EBV positive cases is associated with up regulation of Bob-1 since there is a close association between the expression of Bob-1 and EBV in the present study.

In conclusion, this study confirms the nuclear expression of Bob-1 on H/RS cells in CHL, making the utility of Bob-1 to differentiate between LPHL and CHL difficult. These points open questions concerning environmental factors especially early EBV infection in developing countries and to lesser extent genetic ones. Is there truly a difference between Western and Egyptian CHL, or no differences actually exist as most researchers are more concentrated on their own Western-based populations? We feel that our work necessitates cooperative studies between different countries to answer these questions.

## Abbreviations

H/RS: Hodgkin/Reed-Sternberg, CHL: classic Hodgkin lymphoma, LPHL: lymphocyte predominance Hodgkin lymphoma, LR: lymphocyte rich, MC: mixed cellularity, NS: nodular sclerosis, LD: lymphocyte depletion, EBV: Epstein-Barr virus, EBER ISH: EBV in situ hybridization, LMP-1: EBV latent membrane protein-1

## Competing interests

The author(s) declare that they have no competing interests.
